# Physical and Social Factors Differentiating Acute and Chronic Low Back Pain Among Small- and Medium-Sized Enterprise Workers in Japan: A Cross-Sectional Study

**DOI:** 10.3390/ejihpe16020017

**Published:** 2026-01-27

**Authors:** Yurika Tamekuni, Kenta Okuyama, Atsushi Motohiro, Daijo Shiratsuchi, Minoru Isomura, Linda Abrahamsson, Martin Lindström, Kristina Sundquist, Takafumi Abe

**Affiliations:** 1 Canvas Inc., 1 Hokryou-cho, Matsue City 690-0816, Shimane, Japan; tamekuni@canvas.co.jp (Y.T.); motohiro@canvas.co.jp (A.M.); 2 Graduate School of Social Sciences, Department of Management Studies, Hiroshima University, 1-1-89 Higashi-Senda-machi, Naka-ku, Hiroshima City 730-0053, Hiroshima, Japan; 3 Center for Primary Health Care Research, Department of Clinical Sciences Malmö, Skåne University Hospital, Lund University, Jan Waldenström Gata 35, 20502 Malmö, Sweden; kenta.okuyama@med.lu.se (K.O.); linda.abrahamsson@med.lu.se (L.A.); martin.lindstrom@med.lu.se (M.L.); kristina.sundquist@med.lu.se (K.S.); 4 Center for Community-Based Healthcare Research and Education (CoHRE), Head Office for Research and Academic Information, Shimane University, 223-9 Enya-cho, Izumo City 693-8501, Shimane, Japan; isomura@hmn.shimane-u.ac.jp; 5 Department of Physical Therapy, School of Health Sciences, Faculty of Medicine, Kagoshima University, 8-35-1 Sakuragaoka, Kagoshima City 890-8544, Kagoshima, Japan; shiratsuchi@health.nop.kagoshima-u.ac.jp; 6 Faculty of Human Sciences, Shimane University, 1060 Kawatsu-cho, Matsue City 690-8504, Shimane, Japan

**Keywords:** chronic pain, interpersonal relations, physical load, employees, social support, labor management

## Abstract

Low back pain is common and negatively impacts quality of life and workplace productivity. However, few studies have focused on small- and medium-sized enterprises (SMEs) where risk factors are prevalent. This cross-sectional study examined the association and structural relationships between physical and social factors in low back pain among Japanese SME workers. We analyzed survey data collected between April 2021 and August 2022 from 762 SME workers aged 18–65 years in Shimane, Japan, to assess acute (less than 3 months) and chronic low back pain using a self-reported questionnaire. Workplace physical (physical load) and social (stress of interpersonal relations) factors were measured using a brief job stress-related questionnaire. Associations between workplace factors and acute and chronic low back pain were evaluated using multivariable, multinomial logistic regression. Among the 762 workers, 50.9% and 7.3% reported acute and chronic low back pain, respectively. The odds ratio (OR) for acute pain was 2.08 for ‘Very much so’ in those with a high physical load. Compared with those with low interpersonal stress, the OR for chronic pain was 2.20 for medium stress and 2.82 for high stress. Reducing physical workloads may mitigate acute low back pain, while lowering interpersonal stress at work may reduce chronic low back pain. Future studies should investigate whether improving workplace physical and social factors is longitudinally effective.

## 1. Introduction

Low back pain, a prevalent musculoskeletal disorder, is a leading contributor to disability (GBD 2021 Low Back Pain Collaborators, 2023). Low back pain is associated with reduced health-related quality of life (Yamada et al., 2014) and causes economic losses, such as absenteeism, necessitating preventive measures in workplaces (Nagata et al., 2018). The biopsychosocial model categorizes workplace risk factors for chronic pain into physical (e.g., workload, manual handling), psychological (e.g., job satisfaction, job control), and social factors (e.g., support from superiors, colleague relationships) (Buruck et al., 2019; Cohen et al., 2021; Hoogendoorn et al., 2000; Kraatz et al., 2013; Lang et al., 2012).

Under Japan’s Small and Medium-sized Enterprise Basic Act, small- and medium-sized enterprises (SMEs) are generally defined as companies with a capital of 300 million yen or less, or with 300 or fewer regular employees, although these criteria vary by industry. SMEs account for 99.7% of all businesses in Japan since 2021 (Small and Medium Enterprise Agency, 2021) and play a critical role in the economy. Approximately 70% of the total workforce (50 million workers) are employed in SMEs (Small and Medium Enterprise Agency, 2023). Globally, SMEs employ the majority of the workforce, yet they often have fewer human and financial resources compared to large enterprises. Although several studies have identified workplace social relationships as critical predictors of pain (Christensen & Knardahl, 2010, 2014; Matsudaira et al., 2012, 2014; Tezuka et al., 2022; Unsgaard-Tøndel & Nordstoga, 2022), few have investigated this among SME workers. Little is known about whether workplace social relationships are associated with pain in SMEs, whereas social support may be difficult to obtain and sustain due to lack of human and financial resources. Furthermore, the structural relationship between physical, psychological, and social factors and acute or chronic low back pain has not been well explored in prior analyses. For example, job satisfaction and job control were often adjusted as potential confounders to assess the association between social support and pain (Christensen & Knardahl, 2014; Kawaguchi et al., 2017; Matsudaira et al., 2012, 2014), even though job satisfaction may result from good workplace social support and the absence of pain. Further, understanding acute and chronic pain requires considering both physical and social factors, as their etiologies may differ.

In addition to the limited research on the relationship between working conditions and low back pain in Japanese SMEs, the coronavirus disease 2019 (COVID-19) pandemic significantly altered the working conditions of these enterprises. The pandemic negatively impacted both management practices and workplace environments. At the same time, employees experienced a deterioration in mental health (Tomono et al., 2021), and presenteeism due to low back pain has become a substantial burden (Yoshimoto et al., 2025). Systematic reviews have reported that telework is a contributing factor to musculoskeletal pain (Blank et al., 2023). The transition to telework may also affect the psychosocial aspects of work, such as social connectedness and support from supervisors and colleagues. Furthermore, when job demands are high, this working style transition may aggravate pre-existing adverse health outcomes (He et al., 2021). In this context, investigating the factors contributing to low back pain among workers in the during-COVID-19 era is essential for SMEs to maintain their labor productivity through workplace health initiatives. The experience gained during the COVID-19 pandemic should not be viewed merely as a historical event, but rather as a valuable source of insight for future SME operations, leading to improvements in the workplace environment and the promotion of health initiatives during stable periods as well as during future crises.

Given these research gaps and the evolving landscape for SMEs, we aimed to examine the structural relationship of these factors and assess whether physical factors at work (i.e., physical load) and social factors (i.e., interpersonal relations) were associated with acute and chronic low back pain among Japanese workers at SMEs. Furthermore, we hypothesized that the negative association between physical factors and low back pain could be alleviated by positive social factors, and therefore assessed whether the association between physical load and low back pain was modified by interpersonal relations at work.

## 2. Materials and Methods

### 2.1. Study Participants

We analyzed cross-sectional survey data collected from April 2021 to August 2022 among workers throughout Shimane Prefecture, Japan. The survey, conducted by a healthcare corporation, aimed to assess the current situation of occupational pain. In this study, a priori sample size estimation was not performed. With the cooperation of the Shimane Prefectural Government, we requested surveys from workers in SMEs, which resulted in convenience sampling. When consent was obtained from the heads of these enterprises, we mailed the consent forms and questionnaires and collected responses. This data was collected from workers from 35 SMEs in Shimane Prefecture. The inclusion criteria were obtaining informed consent to participate in the survey and completion of the questionnaire. Data included individuals aged 18–65 years employed full- or part-time. Of 940 respondents, 762 individuals with complete data were included in this analysis.

The study protocol was approved by the Research Ethics Committee for Human Subjects of the Faculty of Human Sciences, Shimane University (Approval number: 2022–2; date of approval: 13 April 2022) and adhered to the Declaration of Helsinki. Informed consent was obtained from all participants.

### 2.2. Outcomes

The definition of chronic pain followed that of the International Association for the Study of Pain (Treede et al., 2019). Participants were asked (Kamada et al., 2014), “Have you had pain in the last year in your lower back?” If the answer was yes, they were further asked, “On how many days have you had this lower back pain?” with options: “Less than 7 days,” “1–4 weeks,” “More than 1 month but less 3 months,” or “More than 3 months.” To classify participants, we categorized outcomes into no pain, acute pain (less than 3 months), and chronic pain.

### 2.3. Explanatory Variables

We used the original 57-item Brief Job Stress Questionnaire (BJSQ), which is the standard instrument for the Japanese National Stress Check Program. Workplace physical and social factors were assessed using the BJSQ developed in Japan (Shimomitsu & Odagiri, 2004). The BJSQ is a validated questionnaire recommended by the Ministry of Health, Labour and Welfare (Shimomitsu, 2000). In SMEs, BJSQ use is proposed as a duty of effort to monitor employee health. The BJSQ demonstrated satisfactory internal consistency, test–retest reliability, and structural validity across the majority of its subscales (Inoue et al., 2014). The use of these items as subscales has been previously reported (Watanabe et al., 2023). Participants were asked whether their jobs required a lot of physical work, with responses recorded as: 1 = not at all, 2 = somewhat, 3 = moderately so, and 4 = very much so. For social factors, participants answered three questions: (1) There are differences of opinion within my department, (2) My department does not get along well with other departments, and (3) The atmosphere in my workplace is friendly (reverse item). Responses in the four categories (as above) were summed and categorized into three groups. The total score for the three questions ranged from 3 to 12, with higher scores indicating higher levels of stress. Scores of 3 to 5 were classified as low, 6 to 7 as medium, and 8 to 12 as high, based on standardized scores from a study of 15,933 male and 8447 female workers in Japan (Shimomitsu & Odagiri, 2004).

### 2.4. Covariates

Gender (male or female), age (continuous), education (high school or more), smoking habit (yes or no), drinking habit (every day/sometimes or hardly), body mass index (continuous), and years of employment (continuous) were considered potential confounders between physical load at work and chronic pain, and were included in the analysis. Directed acyclic graphs (DAGs) were constructed with specialists in social epidemiology, statistics, occupational therapy, physiotherapy, and medicine to identify the adjustment set for these variables. Detailed procedures are provided in Supplemental Figure S1.

### 2.5. Statistical Analysis

All characteristics used in the analyses were described by total and low back pain. Continuous variables were analyzed using the Kruskal–Wallis test, and categorical variables were analyzed using the chi-square test.

Multinomial logistic regression assessed the association between physical load at work or stress of interpersonal relations and acute and chronic low back pain. Models 1 and 2 conducted analyses with each primary explanatory variable (physical load at work or stress of interpersonal relations) entered individually, whereas Model 3 included both primary variables simultaneously. All models were adjusted for confounding factors, including gender, age, educational attainment, smoking habit, drinking habit, years of employment, and body mass index. Interaction terms for physical load and stress of interpersonal relations were included to examine whether the association between physical factors and low back pain is modified by social factors. Physical load and stress of interpersonal relations were treated as continuous variables to account for their ordered nature and increased statistical power, particularly in models with interaction terms.

Several sensitivity analyses were conducted. First, gender and age, as key potential confounders, were examined for residual effects. A squared value of age was included to adjust for non-linear risks of chronic pain, such as exponential or abrupt increases. The interaction between gender and age was also tested. No residual effects or interactions were detected; therefore, gender and age were included as simple terms in the analyses. Second, the job demand-control factor from the theoretical model (Karasek, 1979) was tested alongside the physical load and stress of interpersonal relationships but was not associated with outcomes and was excluded from the final analyses. All statistical analyses were conducted using SPSS Statistics for Windows (version 29.0; IBM Corp., Armonk, NY, USA). A significance level of *p* < 0.05 was established for all tests.

## 3. Results

As shown in Table 1, 388 (50.9%) and 56 (7.3%) of 762 workers reported acute and chronic low back pain, respectively. Older age, low education, longer years of employment, higher physical load, and higher stress of interpersonal relations at work were associated with a higher proportion of low back pain.

Table 2 presents the association between physical load and stress of interpersonal relations at work and low back pain. Compared to the “not at all” group for physical load at work, the “very much so” group (odds ratio [OR] = 2.24, 95% confidence interval [CI] = 1.34, 3.75 in Model 1; OR = 2.08, 95% CI = 1.23, 3.53 in Model 3) showed significantly higher ORs for acute low back pain, but no significant association was observed for chronic low back pain. Compared to the “low” group for the stress of interpersonal relations, the “medium” and “high” group were associated with higher ORs for chronic low back pain in both Model 2 (OR = 2.22, 95% CI = 1.03, 4.75; OR = 3.17, 95% CI = 1.37, 7.36) and Model 3 (OR = 2.20, 95% CI = 1.02, 4.76; OR = 2.82, 95% CI = 1.19, 6.66), respectively. No significant association was observed for acute low back pain. The interaction between physical load and stress of interpersonal relations was not significant for acute (*p* for interaction = 0.71) or chronic low back pain (*p* for interaction = 0.32).

## 4. Discussion

Our findings indicate that physical load was associated with acute low back pain, whereas the stress of interpersonal relations at work was linked to chronic low back pain among workers at SMEs. We noted that the effects of physical load on chronic pain had similar point estimates to acute pain and could potentially be significantly different with a larger sample size. Few studies have examined acute or chronic low back pain in these types of workers from the perspective of physical or social factors. This study provides practical insights for preventing such pain among this population.

According to international trends based on data from the Global Burden of Disease Study 2021, the age-standardized prevalence rate of low back pain per 100,000 population was 9554 globally, and 12,730 in high-income countries (Zhao et al., 2025). In a 2017 study involving 25,000 Japanese workers, the prevalence of low back pain—defined as experiencing pain “often” or “almost always”—was reported to be 23.3% (Sato et al., 2025). A nationwide survey conducted in Japan in 2023 with approximately 2000 participants found that the complaint rate of acute low back pain among individuals aged 20 to 69 ranged from 1.5% to 4.8%, while that of chronic low back pain ranged from 5.1% to 13.7% (Kurita et al., 2024). Although direct comparisons are challenging due to differences in prior studies and assessment methods, the proportion of chronic low back pain in our sample align the range reported in the national survey, whereas the proportion of acute low back pain appeared to be higher than that reported in the national data. In the United States, it has been reported that the prevalence of musculoskeletal disorders decreases as company size increases (Morse et al., 2004). In South Australia, the prevalence of musculoskeletal pain among workers was higher in medium-sized enterprises than in large enterprises (Stewart et al., 2014). These findings suggest that the prevalence of low back pain may be influenced by working conditions, including company size.

Systematic reviews report that heavy physical work is a risk factor for low back pain (da Costa & Vieira, 2010). For example, construction workers with high physical workloads experience awkward postures (e.g., twisting, bending), manual handling of materials, and long working hours, which contribute to musculoskeletal disorders (Anwer et al., 2021). Similarly, repetitive use of body parts among farmers has been linked to low back pain (Lee et al., 2021). Thus, specific work environments with high physical demands are potential risk factors for low back pain. Physical demands at work are suggested to have a stronger association with sick leave for low back pain when assessed subjectively rather than objectively (Petersen et al., 2019). Interventions should ensure no significant load is placed on any body part and verify that workers perceive physical demands as manageable.

Previous research has established that social factors at work—particularly the stress associated with interpersonal relations—are linked to chronic low back pain (Christensen & Knardahl, 2010, 2014; Matsudaira et al., 2012, 2014; Tezuka et al., 2022; Unsgaard-Tøndel & Nordstoga, 2022). This relationship is potentially explained through multidimensional mechanisms connecting psychosocial stress (Lukan et al., 2022; van der Molen et al., 2020), the central nervous system, and low back pain (Knezevic et al., 2021). Moreover, the decision to disclose a health condition to colleagues has been reported to depend on the level of pain experienced (Woticky et al., 2025), and previous work suggests that social support can mitigate the effect of forward bending on low back pain (Villumsen et al., 2016). These findings indicate that higher stress or poor social relationships in work settings may discourage workers from seeking help or addressing their pain. Regarding physical load at work, self-employed workers in physically demanding jobs in the Netherlands reportedly have high motivation and decision-making autonomy, yet economic pressures often prevent them from taking vacations (Cillekens et al., 2024). Such circumstances may contribute to musculoskeletal disorders. Our results suggest that small and medium-sized enterprises (SMEs) in Japan may face difficulties in allowing workers to take leave, particularly when staff numbers are limited. In many Japanese workplaces, pain is often considered “normal,” which may lead to minor pain escalating when social acceptance for acknowledging pain is lacking. Furthermore, although we hypothesized that good interpersonal relations at work might reduce physical load—for example, through colleagues cooperating when lifting heavy objects or taking measures to prevent injuries. This may also apply to office environments, where coworkers may encourage breaks to reduce prolonged sitting. However, we did not observe a significant interaction between physical load and interpersonal stress for either acute or chronic pain.

Our findings highlight the importance of addressing both physical and social factors differently based on the characteristics of low back pain (acute or chronic). For example, introducing machines could reduce the physical load at work, but this is often costly. Physical activities at work can increase stress responses (Abe et al., 2024); therefore, reducing these responses by improving physical functions, such as adding exercises (Ishimaru et al., 2021; Maciel et al., 2018; Tersa-Miralles et al., 2022), is necessary. In Japanese companies, some use radio exercises (radio-taiso) at the start of the workday as a practice. However, it is also crucial to incorporate specialized exercises targeting specific body parts. In addition, a systematic review reported that multidisciplinary interventions are more effective than standard of care in improving pain intensity and functional status in individuals with acute low back pain (Bernaers et al., 2023). From a practical perspective, multidisciplinary interventions based on a comprehensive understanding of the patient may be beneficial.

Despite the insights gained, several limitations warrant consideration. First, the cross-sectional design did not allow us to determine causal associations between physical and social factors and chronic low back pain. While we used DAGs to minimize biases, reverse causation remains possible, e.g., individuals with chronic pain may perceive higher stress in interpersonal relations at work. Second, our study participants were limited to those who responded to the survey at accessible SMEs, and they may not be representative of middle-aged workers at such enterprises. In addition, this study was conducted during the COVID-19 pandemic, from April 2021 to August 2022. Therefore, the findings obtained from this research may have been influenced by the pandemic. For example, remote work and other factors may have restricted communication among workers. Consequently, reduced communication, workplace isolation, and work-family conflicts may have contributed to mental health issues (Artar & Erdil, 2024; Izawa et al., 2022), which could, in turn, be associated with the development of low back pain (Yoshimoto et al., 2025). This limits the generalizability of our data. Third, our outcome, explanatory variables, and potential confounders were subject to potential measurement biases as they were collected via self-reported questionnaires. For instance, individuals under high stress due to interpersonal relations might have reported pain to a greater extent, potentially overestimating the association. Fourth, the sample size for the chronic pain group (only 56 individuals) might have reduced statistical power for all tests, and therefore, this may limit the reliability of the findings. Lastly, residual biases from unmeasured confounders may exist. For example, specific company size (Morse et al., 2004; Stewart et al., 2014) and staff shortages (Kim et al., 2014) potentially influence worker workload and musculoskeletal pain. These factors should be considered in future studies to further clarify this relationship.

## 5. Conclusions

This study found that physical load was associated with acute low back pain, while the stress of interpersonal relations at work was associated with chronic low back pain among workers at SMEs. Addressing physical and social factors in the workplace may effectively reduce the risk of low back pain in this population, but the mechanism may differ between acute and chronic low back pain. Future studies examining the longitudinal association between physical and social factors and low back pain and evaluating interventions through experimental designs are necessary to develop evidence-based strategies.

## Figures and Tables

**Table 1:** Characteristics based on differences in low back pain among workers in Shimane, Japan, 2022.

Characteristic	Overall (*N* = 762)	Low Back Pain			*p* Value
		No (*n* = 318)	Acute (*n* = 388)	Chronic (*n* = 56)	
Age, years ^a¶^	42 (32, 52)	38 (29, 49)	44 (33, 53)	48 (38, 57)	<0.001 ^†^
Gender					0.29 ^‡^
Male	486 (63.8%)	203 (41.8%)	242 (49.8%)	41 (8.4%)	
Female	276 (36.2%)	115 (41.7%)	146 (52.9%)	15(5.4%)	
Education					0.03 ^‡^
High school	333 (43.7%)	121 (36.3%)	185 (55.6%)	27 (8.1%)	
More than high school	429 (56.3%)	197 (45.9%)	203 (47.3%)	29 (6.8%)	
Smoking habit					0.15 ^‡^
Yes	156 (20.5%)	65 (41.7%)	74 (47.4%)	17 (10.9%)	
No	606 (79.5%)	253 (41.7%)	314 (51.8%)	39 (6.4%)	
Drinking habit					0.75 ^‡^
Every day or sometimes	411 (53.9%)	167 (40.6%)	212 (51.6%)	32 (7.8%)	
Hardly (cannot drink)	351 (46.1%)	151 (43.0%)	176 (50.1%)	24 (6.8%)	
BMI, kg/m^2 a¶^	22.0 (20.1, 24.4)	21.8 (20.0, 24.2)	22.4 (20.1, 24.8)	21.9 (20.1, 24.3)	0.19 ^†^
Years of employment ^a¶^	8.0 (3.0, 17.0)	7.0 (3.0, 17.0)	7.5 (3.0, 17.0)	15.0 (5.3, 22.8)	0.01 ^†^
Physical load at work					0.01 ^‡^
Very much so	89 (11.7%)	23 (25.8%)	58 (65.2%)	8 (9.0%)	
Moderately so	170 (22.3%)	62 (36.5%)	96 (56.5%)	12 (7.1%)	
Somewhat	219 (28.7%)	93 (42.5%)	108 (49.3%)	18 (8.2%)	
Not at all	284 (37.3%)	140 (49.3%)	126 (44.4%)	18 (6.3%)	
Stress of interpersonal relations at work					0.02 ^‡^
High	158 (20.7%)	55 (34.8%)	87 (55.1%)	16 (10.1%)	
Medium	360 (47.2%)	145 (40.3%)	185 (51.4%)	30 (8.3%)	
Low	244 (32.0%)	118 (48.4%)	116 (47.5%)	10 (4.1%)	

Note. Continuous variables were analyzed using the Kruskal–Wallis test ^†^, while categorical variables were analyzed using the chi-square test ^‡^. Body mass index = BMI. ^a¶^ Median (interquartile range).

**Table 2:** Multinomial logistic regression analysis of low back pain in relation to “Physical load at work” and “Stress of interpersonal relations at work” among workers in Shimane, Japan, 2022.

Primary Explanatory Variables		Low Back Pain											
		Acute						Chronic					
		Model 1		Model 2		Model 3		Model 1		Model 2		Model 3	
		OR	95% CI	OR	95% CI	OR	95% CI	OR	95% CI	OR	95% CI	OR	95% CI
Physical load at work	Not at all	1.00	Ref			1.00	Ref	1.00	Ref			1.00	Ref
	Somewhat	1.21	0.83–1.75			1.21	0.83–1.77	1.18	0.58–2.41			1.09	0.52–2.28
	Moderately so	1.46	0.98–2.19			1.38	0.92–2.08	1.21	0.55–2.68			1.02	0.45–2.33
	Very much so	2.24	1.34–3.75			2.08	1.23–3.53	2.13	0.84–5.37			2.07	0.80–5.36
Stress of interpersonal relations at work	Low			1.00	Ref	Ref	-			1.00	Ref	Ref	-
	Medium			1.28	0.92–1.79	1.26	0.89–1.78			2.22	1.03–4.75	2.20	1.02–4.76
	High			1.49	0.98–2.27	1.42	0.92–2.17			3.17	1.37–7.36	2.82	1.19–6.66
*p* for interaction ^§^						0.71						0.32	

Note. Models 1 and 2 conducted multinomial logistic regression analyses with each primary explanatory variable entered individually, while Model 3 included both primary variables simultaneously. All models were adjusted for confounding factors, including gender, age, educational attainment, smoking habit, drinking habit, years of employment, and body mass index. ^§^ *p* for interaction reflects the results of incorporating the combined variable of physical load at work and stress of interpersonal relations at work. CI: confidence interval; OR: odds ratio.

## Data Availability

The datasets generated during and/or analyzed during the current study are available from the corresponding author upon reasonable request.

## Supplementary Materials

The following supporting information can be downloaded at https://www.mdpi.com/article/10.3390/ejihpe16020017/s1. Figure S1: Determined covariates (sex, age, educational attainment, smoking habit, drinking habit, years of employment, and body mass index) using Directed Acyclic Graphs (DAGs).

## Abbreviations

The following abbreviations are used in this manuscript:

BJSQ	Brief Job Stress Questionnaire
CI	Confidence interval
COVID-19	Coronavirus disease 2019
DAGs	Directed acyclic graphs
OR	Odds ratio
SMEs	Small- and medium-sized enterprises
