# Supplementary figures and images for: Physical and Social Factors Differentiating Acute and Chronic Low Back Pain Among Small- and Medium-Sized Enterprise Workers in Japan: A Cross-Sectional Study
Source: Eur J Investig Health Psychol Educ. 2026 Jan 27;16(2):17. doi: 10.3390/ejihpe16020017 (PMC12939500; doi:10.3390/ejihpe16020017)

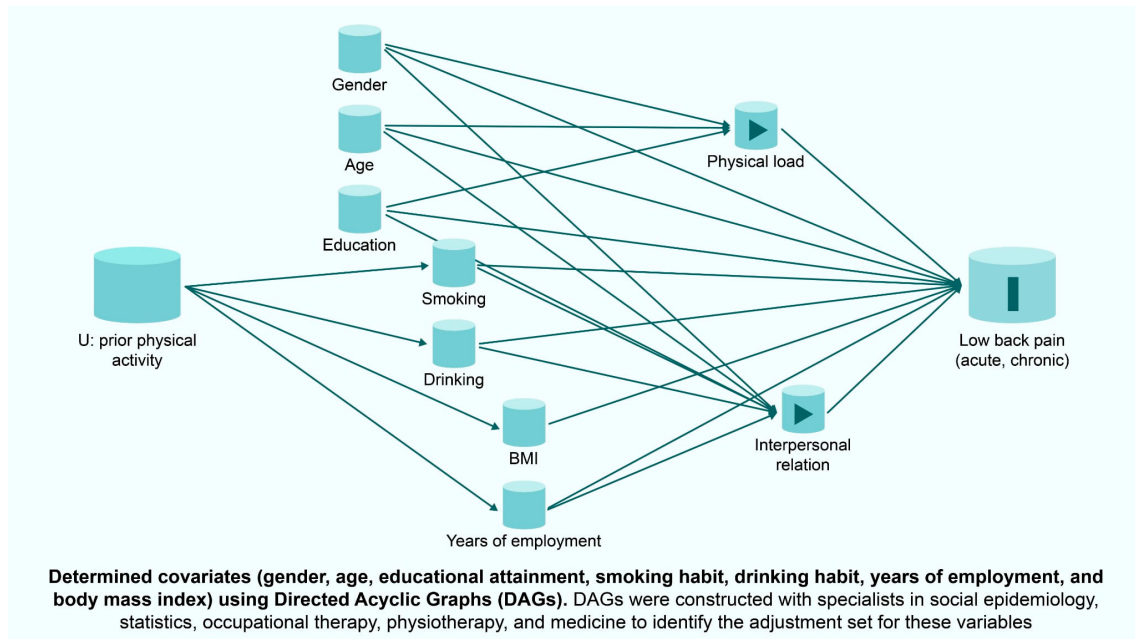

Figure S1. Directed acyclic graphs (DAGs) for covariate selection.

Supplement: Supplementary file 1 [file ejihpe-16-00017-s001.zip › ejihpe-4085971-supplementary.pdf]
